# Association of Hair Concentrations of Antiretrovirals with Virologic Outcomes Among People Living with HIV in Guangxi, China

**DOI:** 10.2147/PPA.S277965

**Published:** 2021-04-23

**Authors:** Quan Zhang, Xiaoming Li, Shan Qiao, Shuaifeng Liu, Zhiyong Shen, Yuejiao Zhou

**Affiliations:** 1 South Carolina SmartState Center for Healthcare Quality (CHQ), Arnold School of Public Health, University of South Carolina, Columbia, SC, USA; 2 Institute of Pedagogy and Applied Psychology, School of Public Administration, Hohai University, Nanjing, Jiangsu, People’s Republic of China; 3 Guangxi Zhuang Autonomous Region Center for Disease Control and Prevention, Nanning, Guangxi, People’s Republic of China

**Keywords:** hair concentrations, antiretroviral, viral load, people living with HIV

## Abstract

**Background:**

Hair concentrations of antiretrovirals are an innovative and non-invasive method for measuring cumulative antiretroviral exposure and assessing long-term antiretroviral adherence. This study aimed to examine hair concentrations of antiretrovirals in relation to virologic outcomes among PLHIV in Guangxi, China.

**Methods:**

Cross-sectional data of hair concentrations of antiretrovirals and HIV viral load were collected from 215 PLHIV in Guangxi, China. Multivariate logistic regression analyses were used to examine the association of hair concentrations of antiretrovirals with virologic outcomes.

**Results:**

Of the 215 participants, 215, 67, and 163 PLHIV are receiving lamivudine, zidovudine, and efavirenz, respectively. Multivariate analysis revealed that hair concentrations of lamivudine [odds ratio = 16.52, 95% CI 2.51–108.60, *p* = 0.004] and efavirenz [odds ratio = 14.26, 95% CI 1.18–172.01, *p* = 0.036], but not zidovudine [odds ratio = 1.77, 95% CI 0.06–56.14, *p* = 0.747], were the strongest independent predictor of virologic suppression when controlling for sociodemographic and other HIV-related characteristics.

**Conclusion:**

Hair concentrations of lamivudine and efavirenz were the strongest independent predictor of virologic suppression among Chinese PLHIV. Hair analysis of antiretrovirals may provide a non-invasive, cost-effective tool that predicts virologic suppression among PLHIV in China.

## Introduction

Combination antiretroviral therapy (cART) is the primary modality for treating and preventing HIV infection and can substantially reduce HIV-related morbidity, mortality, and transmission.^1^,^2^ Optimal cART adherence is a critical determinant for adequate antiretroviral exposure, which has been vital to virologic suppression and improved clinical outcomes among people living with HIV (PLHIV).^3–5^ Given the pharmacological relationship between cART adherence and antiretroviral exposure, analysis of antiretroviral concentrations in biological samples has been used as an objective method for measuring antiretroviral exposure and assessing antiretroviral adherence. Pharmacologic markers of antiretroviral exposure and antiretroviral adherence often involve measurement of antiretroviral concentrations in biological samples, such as plasma,^6^,^7^ urine,^8^ peripheral blood mononuclear cells (PMBC),^9^ dried blood spots (DBS),^10^,^11^ and hair.^12^,^13^ Among those metrics, plasma and urine levels can only measure real-time or short-term antiretroviral exposure and antiretroviral adherence (eg, hours to days). In addition, plasma and urine antiretroviral levels are susceptible to day-to-day variation.^4^,^14^ Until now, DBS levels can measure cumulative exposure to several antiretrovirals that are activated intracellularly^4^,^15^ and longer-term antiretroviral adherence (eg, days to weeks). Alternatively, hair levels can measure cumulative exposure to all antiretrovirals^4^,^16^,^17^ and long-term antiretroviral adherence (eg, weeks to months) because hair levels reflect antiretroviral uptake from the systemic circulation over weeks to months. In addition, hair collection is non-invasive and requires minimal training, and hair specimens can be stored and shipped at room temperature without biohazardous precautions.^18^,^19^ All of these advantages make hair concentrations of antiretrovirals appealing as an innovative method for measuring cumulative antiretroviral exposure and assessing long-term antiretroviral adherence.

Presently, there are more than 25 antiretrovirals in multiple classes approved by US Food and Drug Administration for HIV treatment, such as nucleoside reverse transcriptase inhibitors (NRTI), non-nucleoside reverse transcriptase inhibitors (NNRTI), protease inhibitors (PI), and integrase strand transfer inhibitor (INSTI, eg, raltegravir).^20^ Worldwide, recommended cART is consisting of two or more classes of antiretrovirals. For example, currently, WHO recommends one INSTI (eg, dolutegravir) and two NRTIs [eg, lamivudine (3TC), zidovudine (AZT), tenofovir (TFV)] as the first-line treatment, one NNRTI [eg, efavirenz (EFV), nevirapine (NVP)] and two NRTIs as alternative first-line treatment, and one PI [eg, atazanavir (ATV) and lopinavir/ritonavir (LPV/RTV)] and two NRTIs as the second-line treatment. As evidenced by a comprehensive review,^4^ an ever-increasing body of literature has shown that more than ten hair concentrations of antiretrovirals were employed as biomarkers of cumulative antiretroviral exposure and/or long-term antiretroviral adherence to predict virologic outcomes in Africa, North America, and Europe settings. Although China rolled out its free cART project for HIV treatment in 2004, and access to first- or second-line cART has been scaled up across the country, limited data are available on the association of hair concentrations of antiretrovirals with virologic outcomes among Chinese PLHIV. Furthermore, the simultaneous determination of hair concentrations of antiretrovirals in multiple classes is technically possible.^21–24^ However, major of the previous studies only investigated the association of hair concentrations of a single antiretroviral^13^,^25–33^ or antiretrovirals in a single class^34^ with virologic outcomes. Limited data are available on the association of hair concentrations of antiretrovirals in multiple classes with virologic outcomes.

Accordingly, we present this analysis of hair concentrations of three antiretrovirals (3TC, AZT, and EFV) in two classes (NRTI and NNRTI) in relation to virologic outcomes among Chinese PLHIV.

## Materials and Methods

### Participants

The participants of this study were recruited from Wave 3 of the HIV disclosure project, which is a longitudinal study that aims to explore the mechanism underlying the linkage between HIV disclosure and clinical outcomes in Guangxi, China.^35–37^ With the Guangxi Center for Disease Prevention and Control (Guangxi CDC) ‘s assistance and collaboration, we randomly selected 10 clinic sites with the largest number of HIV/AIDS cases from 17 cities and 75 counties in Guangxi. The recruitment procedure, including inclusion and exclusion criteria, was described in the previous studies.^35^ Briefly, medical staff or HIV case managers at the study sites referred potential participants to the research team members. Research team members screened PLHIV for eligibility, discussed the benefits and risks of the study, and invited them to participate. Research team members were local CDC staff or healthcare workers in the HIV clinics who had received intensive training on research ethics and interview skills with PLHIV before the field data and hair specimen collection. Finally, a total of 446 PLHIV participated in Wave 1 of the HIV disclosure project, and 428 PLHIV participated in Wave 3 of the HIV disclosure project.

This study followed the Declaration of Helsinki and was approved by ethical review boards of the University of South Carolina (Columbia, USA) and the Guangxi Zhuang Autonomous Region Center for Disease Prevention and Control (Nanning, China). The written informed consent also has been obtained from the participants.

### Hair Sample Collection and Assay

Hair samples were cut from the posterior vertex region as close as possible to the scalp following a standard protocol^38^,^39^ after participants finished the survey. The hair sample (about 1 cm of the proximal section) was rinsed with methanol and dried under a blow of pure nitrogen gas. Then, hair sample was chopped to 1–2 mm length segments with scissors, and 10 mg weighed, processed, and analyzed using high-performance liquid chromatography (Agilent 1200 HPLC system, Agilent, Waldbronn, Germany) and tandem mass spectrometry (ABI 3200Qtrap, ABI, Foster City, CA, USA) (LC-MS/MS) as described in previous studies.^21^,^24^,^40^ Briefly, following the 2013 FDA guidelines, antiretrovirals in the cut hair samples were extracted with methanol and internal standard in a 37°C shaking water bath overnight (>16 hours) and then analyzed by an LC-MS/MS. Standard curves were linear in the range of 6–40,000 pg for 3TC, 10–40,000 pg for AZT, and 12–40,000 pg for EFV with good linearity and reproducibility. The relative error (%) and precision [coefficients of variation] for spiked quality control hair samples at low, medium, and high concentrations were all <13%. The recoveries at low, medium, and high concentrations were all ≥91.1%. The lower limit of quantitation was 6 pg/mg, 10 pg/mg, and 12 pg/mg for 3TC, AZT, and EFV.

### Sociodemographic and HIV-Related Characteristics

Participants provided information on their sociodemographic characteristics, including age (years), gender (male vs female), ethnicity (Han vs non-Han), marital status (married vs other), education level (>9 years vs ≤9 years), employment status (employed vs unemployed), monthly household income level (≥2000 Chinese Yuan vs <2000 Chinese Yuan).

The participants’ HIV viral load and other HIV-related characteristics at the time of hair collection, including date of HIV diagnosis, current cART status, date of starting current cART, and CD4 count, were extracted from their clinical records. Given that the “Undetectable = Untransmittable” initiative defined undetectable viral load as viral load less than 200 copies/mL that cannot sexually transmit HIV to others,^41^ we defined virologic suppression as viral load less than 200 copies/mL, and virologic failure as viral load equal or more than 200 copies/mL. Years of HIV diagnosis and current cART referred to the time period from the initial date of confirmed HIV diagnosis and the start date of current cART to the date of hair collection, respectively. The cART status was category into first-line and second-line cART. CD4count was dichotomized as ≥350 cells/mm^3^ vs <350 cells/mm^3^ because free cART was offered to PLHIV, whose CD4 count was less than 350 cells/mm^3^.

Self-reported adherence was determined by asking participants to indicate how often they took their antiretrovirals as prescribed in the past month. Response options ranged from 1 (100% of the time) to 5 (I have not taken any of my prescribed medications). This measure has been found to predict CD4+ T cell counts and viral load as well as, or better than, other measures of self-reported adherence.^42^ Given that self-reported data are generally skewed and overestimated, we dichotomized self-reported adherence to 100% and less than 100%.^36^

### Statistical Analysis

Of 428 PLHIV, 213 were excluded from the final analysis because they had not yet started cART (n=13) or stopped cART (n=2) or unavailable HIV viral load (n=198), leaving an effective sample of 215 participants for analysis.

Descriptive statistics were used to summarize hair concentrations of antiretrovirals, sociodemographic, and HIV-related characteristics. Categorized data were expressed as number (n) and percentage (%). A one-sample Shapiro–Wilks test was used to examine normally distributed data. Non-normally distributed data were expressed as the median and interquartile range (IQR), and normally distributed data were expressed as mean (M) and standard deviation (SD). Chi-square test (for categorical variables), *t*-test (for normally distributed continuous variables), and Mann–Whitney *U*-test (for non-normally distributed continuous variables) were used to compare differences between the virologic suppression and virologic failure groups in terms of the sociodemographic, hair concentrations of antiretrovirals, and other HIV-related characteristics. Spearman correlation analysis was performed to examine the association among hair concentrations of antiretrovirals and the association of hair concentrations of antiretrovirals with sociodemographic and other HIV-related variables.

Multivariate linear regression analysis was used to check for collinearity, and all variables showed good tolerance (>0.1) and variance inflation factors (<10). Logistic regression analysis was used to estimate the association of hair concentrations of antiretrovirals (3TC, AZT, and EFV separately) with the dichotomous outcome of virologic response. Hair concentrations of 3TC, AZT, and EFV tertiles were calculated, and the second and third tertiles were contrasted with the first tertile as the reference category. The univariate logistic regression model was used to examine those associations. To fully control the potential impact of sociodemographics and other HIV-related variables on virologic response, the multivariate logistic regression model with the enter method was used to examine those associations adjusting sociodemographics and other HIV-related variables. Crude odds ratios (cORs) and their 95% confidence interval (95% CI) were reported for univariate analysis, and adjusted odds ratios (aORs) and their 95% CI were reported for multivariate analysis. All data analyses were performed using SPSS 26.0 (SPSS Inc., Chicago, IL).

## Results

Of the 215 PLHIV with a mean (SD) age of 41 (8) years (Table 1), 68.4% were male, 55.7% were Han ethnicity, 78.6% were married, 86.5% were employed, 86% were receiving the first-line cART, 84.2% were reporting 100% cART adherence, 52.6% were achieving CD4 count- ≥ 350 cells/mm^3^, and 91.6% were achieving virologic suppression. Most of the sample had low levels of education and income, with 96.1% reporting not complete junior high school and 54% reporting a monthly household income of less than 2000 Chinese Yuan (or approximately US$300 during the time of the study). The median years of HIV diagnosis and current cART were 2.8 years and 2.7 years. Significant differences between virologic suppression and virologic failure groups were only found in current cART status (*p* = 0.013) and CD4 count (*p* = 0.007).

**Table 1:** Sociodemographic and HIV-Related Characteristics of Participants

Characteristics	Total n=215 (100%)	Virologic Suppression n=197 (91.6%)	Virologic Failure n=18 (8.4%)	p-value
Age, years	43.13 ± 8.31	43.44 ± 11.71	43.10 ±7.97	0.865
Gender				
Male	147 (68.4%)	137 (69.5%)	10 (55.6%)	0.222
Female	68 (31.6%)	60 (30.5%)	8 (44.4%)	
Ethnicity				
Han	122 (56.7%)	113 (57.4%)	9 (50.0%)	0.546
No-Han	93 (44.3%)	84 (42.6%)	9 (50.0%)	
Marital status				
Married	169 (78.6%)	157(79.7%)	12 (66.7%)	0.197
Others	46 (21.4%)	40 (20.3%)	6 (33.3%)	
Education levels				
> 9 years	18 (8.4%)	18 (9.1%)	0 (0%)	0.180
≤ 9 years	197 (91.6%)	179 (90.9%)	18 (100%)	
Employment status				
Employed	186 (86.5%)	172 (87.3%)	14 (77.8%)	0.257
Unemployed	29 (13.5%)	25 (12.7%)	4 (22.2)	
Monthly income levels				
≥2000 Chinese Yuan	99 (46.0%)	92 (46.7%)	7 (38.9%)	0.524
<2000 Chinese Yuan	116 (54.0%)	105 (53.3%)	11 (61.6%)	
Years of HIV diagnosis, years	2.8 (2.3–3.4)	2.8 (2.3–3.4)	2.5 (1.9–3.3)	0.228
Current cART status				
First-line cART	185 (86.0%)	173 (87.8%)	12 (66.7%)	0.013
Second-line cART	30 (14.0%)	24 (12.2%)	6 (33.3%)	
Years of current cART, years	2.7 (2.3–3.4)	2.8 (2.3–3.4)	2.5 (1.9–3.2)	0.249
CD4 count-, cells/mm^3^				
< 350	102(47.4%)	88 (44.7%)	14 (77.8%)	0.007
≥ 350	113(52.6%)	109 (55.3%)	4 (22.4%)	
Self-reported adherence				
100%	181 (84.2%)	167 (93.2%)	14 (77.8%)	0.436
Less than 100%	34 (15.8%)	30 (15.2%)	4 (22.2%)	

**Abbreviation:** cART, combination antiretroviral therapy.

As shown in Table 2, marginal or significant correlation was found between hair 3TC concentrations and gender (*p* = 0.097), education levels (*p* = 0.080), employment status (*p* = 0.013), current cART status (*p* = 0.063), years of current cART (*p* = 0.096) and self-reported adherence (*p* = 0.008), between hair AZT concentrations and monthly income levels (*p* = 0.024), current cART status (*p* = 0.045), self-reported adherence (*p* = 0.005) and CD4 count (*p* = 0.097), and between hair EFV concentrations and self-reported adherence (*p* = 0.057). In addition, positive correlations were found between hair 3TC concentrations and hair AZT concentrations (n = 68, *r* = 0.465, *p* < 0.001), between hair 3TC concentrations and hair EFV concentrations (n = 163, *r* = 0.414, *p* < 0.001), and between hair AZT concentrations and hair EFV concentrations (n=38, *r* = 0.597, *p* < 0.001).

**Table 2:** Correlation Between Hair Concentrations of Antiretrovirals and Sociodemographic and Other Clinical Characteristics

Characteristics	3TC Concentrations N=215		AZT Concentrations N=67		EFV Concentrations N=163	
	*r*	p-value	*r*	p-value	*r*	p-value
Age	0.100	0.142	0.026	0.836	0.005	0.947
Gender	0.114	0.097	0.188	0.127	0.127	0.107
Ethnicity	−0.034	0.662	0.123	0.321	0.083	0.293
Marital status	−0.050	0.468	0.022	0.863	−0.089	0.259
Education levels	0.120	0.080	0.043	0.730	−0.023	0.770
Employment status	−0.170	0.013	−0.130	0.294	−0.028	0.724
Monthly income levels	0.112	0.102	0.193	0.024	0.031	0.693
Years of HIV diagnosis	0.105	0.125	−0.097	0.435	−0.126	0.108
Current cART satus	0.127	0.063	−0.245	0.045	-	-
Years of current cART	0.114	0.096	−0.097	0.435	−0.090	0.252
Self-reported adherence	0.181	0.008	0.340	0.005	0.149	0.057
CD4 count	−0.016	0.811	0.240	0.097	−0.104	0.186

**Abbreviations:** cART, combination antiretroviral therapy; 3TC, lamivudine; AZT, zidovudine; EFV, efavirenz.

As shown in Table 3, of the 215 participants, 215, 67, and 163 PLHIV are receiving 3TC, AZT, and EFV, and 197, 60, and 153 PLHIV are achieving virologic suppression, respectively. PLHIV with virologic suppression had significant higher hair concentrations of 3TC, AZT, and EFV than those with virologic failure (*p* = 0.008, *p* = 0.016, and *p* = 0.030, respectively). The median and interquartile range (IQR) of hair concentrations of 3TC, AZT, and EFV among those with virological suppression was 476.55 (315.33–695.38) pg/mg, 289.48 (166.35–471.21) pg/mg, and 4814.74 (3472.07–7111.86) pg/mg, respectively. The value of 315.33 pg/mg, 166.35 pg/mg, and 3472.07 pg/mg (lower IQR for those with virological suppression) defined a cut-off below which most participants experienced virological failure.

**Table 3:** Hair Concentrations of Antiretrovirals by the Virologic Outcome

Antiretrovirals	Virologic Suppression		Virologic Failure		p-value
	n	Median (IQR), pg/mg	n	Median (IQR), pg/mg	
3TC	197	476.55 (315.33–695.38)	18	247.42 (36.66–454.67)	0.008
AZT	60	289.48 (166.35–471.21)	7	10.00 (10.00–185.98)	0.016
EFV	153	4814.74 (3472.07–7111.86)	10	3262.33 (53.18–5252.53)	0.030

**Abbreviations:** IQR, interquartile range; 3TC, lamivudine; AZT, zidovudine; EFV, efavirenz.

**Table 4:** Logistic Regression Results Predicting Virologic Suppression Based on Hair Concentrations of Antiretrovirals

		Univariate		Multivariate	
	n (%)	cORs (95% CI)	p	aORs (95% CI)	*p*
3TC (n=215)					
Lowest tertile	60 (84.5%)	1.00 (ref)	–	1.00(ref)	–
Middle tertile	67 (94.4%)	3.07 (0.93–10.16)	0.066	5.65 (1.21–26.34)	0.028
Highest tertile	70 (95.9%)	4.28 (1.14–16.05)	0.031	16.52 (2.51–108.60)	0.004
AZT (n=67)					
Lowest tertile	17 (77.3%)	1.00 (ref)	–		
Middle tertile	21 (95.5%)	6.18 (0.66–58.03)	0.111	1.06 (0.02–47.85)	0.976
Highest tertile	22 (95.7%)	6.47 (0.69–60.68)	0.102	1.77 (0.06–56.14)	0.747
EFV (n=163)					
Lowest tertile	49 (90.7%)	1.00 (ref)	–		
Middle tertile	50 (92.6%)	1.28 (0.32–5.02)	0.728	1.72 (0.34–8.78)	0.511
Highest tertile	54 (98.2%)	5.51 (0.62–48.82)	0.125	14.26 (1.18–172.01)	0.036

**Abbreviations:** CI, confidence interval; cOR, crude odds ratio; aOR, adjusted odds ratio; 3TC, lamivudine; AZT, zidovudine; EFV, efavirenz.

The results of logistic regression models of virologic suppression are presented in Table 4. The cORs were 4.28 [95% CI 1.14–16.05, *p*=0.031], 6.47 [95% CI 0.69–60.68, p=0.102], and 5.51 [95% CI 0.62–48.82, p=0.125] for those with hair concentrations of 3TC, AZT, and EFV in the highest tertile compared to the lowest tertile in univariate analysis. The aORs were 16.52 [95% CI 2.51–108.60, p=0.004], 1.77 [95% CI 0.06–56.14, p=0.747], and 14.26 [95% CI 1.18–172.01, p=0.036] for those with hair concentrations of 3TC, AZT, and EFV in the highest quartile compared to the lowest quartile when controlling for sociodemographic and other HIV-related characteristics.

## Discussion

Our study is the first to examine the association of hair concentrations of various antiretrovirals with virologic outcomes in a cohort of Chinese PLHIV on cART. We show that, while hair concentrations of 3TC, AZT, and EFV were significantly higher in PLHIV with virologic suppression than those with virologic failure, hair concentrations of 3TC and EFV, but not AZT, are the strongest independent predictor of virologic suppression when controlling for sociodemographic, and other HIV-related characteristics. In addition, we found positive correlations among hair concentrations of antiretrovirals and between self-reported adherence and hair concentrations of antiretrovirals.

Our results regarding PLHIV with virologic suppression had higher hair concentrations of 3TC, AZT, and EFV than those with virologic failure were in line with previous studies employing hair concentrations of 3TC,^13^ EFV,^12^,^43^ TFV,^33^ NVP,^12^ LPV,^12^,^29^,^31^,^34^,^43^ RTV,^12^,^29^,^34^ ATV^34^,^44^ and indinavir.^25–27^ Interestingly, our study revealed that hair concentrations of 3TC and EFV, but not AZT, are the strongest independent predictor of virologic suppression. Our findings of hair concentrations of 3TC and EFV added evidence to existing studies that reported that hair concentrations of antiretrovirals (eg, TFV,^33^ EFV,^43^,^45^ NVP,^30^ LPV,^31^,^32^,^34^ ATV^28^,^34^) were the strongest independent predictor of virologic suppression. Regarding the nonsignificant finding of hair AZT concentrations, the small sample size and lower hair AZT concentrations could contribute to this finding. As seen in Table 3, among 215 PLHIV on cART, only 67 PLHIV were receiving AZT-based cART, and hair AZT concentrations were much lower than hair concentrations of 3TC and EFV.

We found a positive correlation between self-reported adherence and hair concentrations of 3TC, AZT, and EFV, which was partly in line with previous studies by using hair concentrations of antiretrovirals (eg, ATV^28^ and TFV^36^). This finding might also indicate that our study’s adherence measure could be better detection of cART adherence.^42^ In addition, we found positive correlations among hair concentrations of 3TC, AZT, and EFV, which might indicate a significant drug–drug interaction among those antiretrovirals. Moreover, we found that hair 3TC concentrations were easily affected by sociodemographic and HIV-related characteristics than hair concentrations of AZT and EFV did (Table 2), which added not only the understanding of the factors related to hair concentrations of antiretrovirals,^19^,^37^ but also the potential reason that hair EFV concentrations had higher effect than hair 3TC concentrations, in addition to hair EFV concentrations are much higher than hair 3TC concentrations.

Our study has several limitations. First, the current study was based on cross-sectional data, which prevents making causal inferences. Second, the current study only employed hair concentrations of three antiretrovirals. Future studies should examine some other commonly used free antiretrovirals (eg, TFV, NVP, and LPV/RTV) in a large sample size with a more innovative methodology (eg, machine learning). Third, we employed the lower IQR of hair concentrations of antiretrovirals for those with virologic- suppression as the recommended cut-offs for viral failures based on the Chawana et al.^44^ Future studies should determine such cut-offs using pharmacokinetic modeling.^46^ Fourth, because all participants were from Guangxi, China, these findings may not be generalizable to PLHIV in other settings. Future research should employ both Chinese PLHIV and PLHIV in other countries to examine the association between hair concentrations of antiretrovirals with virologic outcomes. Finally, data were not available in the current study on some factors (eg, illegal drugs use) that might influence virologic outcomes or hair concentrations of antiretrovirals.^19^ Those factors should be considered in future research.

## Conclusion

In summary, our findings indicated that hair concentrations of antiretrovirals were significantly associated with virologic outcomes among Chinese PLHIV. Hair analysis of antiretrovirals may provide a non-invasive, cost-effective tool that predicts virologic suppression among Chinese PLHIV.
